# Effect of Fiber and Metal Reinforcement on the Flexural Properties of Printed and Conventional Provisional Restorative Materials

**DOI:** 10.3390/polym18121546

**Published:** 2026-06-22

**Authors:** João Carlos Ramos, Gabriela Almeida, Francisco Silva, Neila Gani, Ana Messias, Alexandra Vinagre

**Affiliations:** 1 Institute of Operative Dentistry, Faculty of Medicine, University of Coimbra, 3000-075 Coimbra, Portugal; jcramos@fmed.uc.pt (J.C.R.); avinagre@fmed.uc.pt (A.V.); 2 Center for Innovation and Research in Oral Sciences (CIROS), Faculty of Medicine, University of Coimbra, 3000-075 Coimbra, Portugal; 3 Institute of Prosthodontics and Oral Implantology, Faculty of Medicine, University of Coimbra, 3000-075 Coimbra, Portugal; galmeida@uc.pt; 4 Dentistry Department, Faculty of Medicine, University of Coimbra, 3000-075 Coimbra, Portugal; franciscoribeirodasilvamd@gmail.com (F.S.); neygani13@gmail.com (N.G.); 5 Center of Mechanical Engineering Materials and Processes (CEMMPRE), University of Coimbra, 3030-788 Coimbra, Portugal

**Keywords:** prosthodontics, provisional restorative materials, 3D printing, fiber reinforcement, mechanical properties, flexural strength, flexural modulus

## Abstract

(1) Background: Provisional restorations play a crucial role in maintaining oral function and must exhibit adequate mechanical properties, particularly fracture resistance, to ensure structural integrity throughout the provisional phase. The aim of this study was to compare the flexural strength and modulus of materials used for provisional dental prostheses, with and without fiber or metal reinforcement. (2) Methods: Standardized specimens (2 × 2 × 25 mm) were fabricated from an acrylic resin (Unifast LC), a 3D-printed resin (NextDent C&B), and a bis-acryl resin (Luxatemp Fluorescence). For each material, four experimental subgroups were established: no reinforcement, two types of glass fiber reinforcement (EverStick C&B and EverStick Post NET), and metal reinforcement. Specimens were subjected to a three-point bending test. Flexural strength and flexural modulus were analyzed using a two-way, non-parametric ANOVA with the aligned rank transform. The significance level was set at 0.05. (3) Results: Material type and reinforcement strategy significantly affected flexural strength and flexural modulus. Fiber reinforcement with EverStick C&B yielded the highest values across all materials, particularly in the acrylic resin. Metal reinforcement showed moderate improvements, whereas EverStick NET had limited or no effect and reduced strength in the bis-acryl resin. Reinforced specimens exhibited altered fracture behavior, preventing complete separation after failure. (4) Conclusions: Fiber reinforcement, particularly with EverStick C&B, significantly enhances the flexural strength and modulus of provisional materials. The reinforcement performance is dependent on its type and material interaction, modifying fracture behavior by preventing complete separation.

## 1. Introduction

Provisional restorations are paramount elements in fixed prosthodontics, ensuring the preservation of masticatory function, aesthetics, and health of dental and periodontal tissues until the definitive prosthesis is cemented [1,2,3]. Appropriate properties must be ensured, allowing integrity and patient comfort [1,4].

Regarding composition, temporary materials can be classified as acrylic resins, based on polymethyl methacrylate (PMMA) or polymethyls/butyl methacrylate (PEMA), or bis-acrylic composite resins, formulated with multifunctional dimethacrylates, such as bisphenol A-glycidyl methacrylate (Bis-GMA) and urethane dimethacrylate (UDMA) [2,3,5,6]. While PEMA has been associated with unacceptable marginal adaptation, hindering long-term clinical success [7], PMMA has been widely employed due to the satisfactory mechanical properties and color stability [2,3,4]. Nevertheless, exothermic reaction during polymerization, high polymerization shrinkage, and monomer release were presented as disadvantages of the latter [3,8]. Alternatively, bis-acrylic resins are often preferred for their superior aesthetics, ease of handling, and shorter working times [8,9].

Despite their widespread use, these materials may exhibit clinical limitations, such as low flexural strength, fracture risk under masticatory loads, and dimensional instability, particularly in long-term or extensive rehabilitation cases [4,7,9]. Such limitations have driven the development of alternative systems aimed at enhancing clinical performance [5].

Technological and scientific advances led to the development of new techniques that have revolutionized the field of prosthodontics, such as the creation of CAD/CAM systems, which have enabled a faster and more efficient approach [2]. Numerous disadvantages have been reported for subtractive milling techniques, such as significant material waste, potential wear of milling bits, and reduced marginal adaptation, particularly for complex restorations, as this is determined by the movement, type, and size of the used tool [5,10].

In an attempt to overcome these constraints, 3D printing has emerged as a promising technology for producing provisional structures [5,11]. This additive manufacturing technique presents numerous advantages, including enhanced adaptation and precision, while offering fast production with reduced material waste [2,3,5,12]. Nevertheless, it is noteworthy that the higher cost of materials and equipment and the superior polymerization shrinkage and surface roughness result from the deposition of several resin layers [13,14,15,16,17]. Methods for the fabrication of printed materials include stereolithography, digital light processing, selective laser sintering, fused deposition modeling, polyjet, and bioprinting [2,5]. These methods allow the production of both temporary and definitive restorations, surgical guides, and definitive models. Factors to consider include the post-curing technique, post-polymerization and temperature, thickness of the printed layer, shrinkage between the layers, curing speed, intensity, angle, and printing direction, as each of them influences the mechanical properties of 3D printed materials [5,18]. Mechanical performance is highly dependent on all these factors [13,14,15,16,17].

It is noteworthy that suitable materials must be selected for each restoration, particularly considering their biological, aesthetic, and mechanical properties, which will allow the restoration to withstand the occlusal forces of mastication without fracture or displacement [3,5,19]. As provisional restorations are particularly prone to fracture, various reinforcement approaches have been proposed to improve their mechanical performance and, consequently, guarantee adequate function during interim phases. Evidence from the literature suggests that reinforcement can significantly improve the durability of these restorations by decreasing the likelihood of chipping, surface wear, and catastrophic fracture over time [20].

With the purpose of addressing provisional restorations’ mechanical limitations, different strategies have been suggested, including reinforcement with fiber or a stainless steel wire [7,21]. The efficacy of the former depends on factors such as fiber type, orientation, length, degree of impregnation, and adhesion to the polymer matrix [22,23]. The literature yields heterogeneous results regarding the different available materials for provisional restorations, thereby precluding a scientifically accurate determination of which materials have the most favorable characteristics for temporary rehabilitation [2].

Thus, the aim of this study was to evaluate and compare the flexural strength and modulus of a 3D-printed composite resin, a PMMA-based resin, and a bis-acrylic resin, with and without reinforcement, and with metal and two types of glass fiber, for temporary restorations. The tested null hypotheses in this study were that (1) there is no difference between the flexural strength and modulus of the tested materials, (2) the use of reinforcement has no influence on the flexural strength and modulus, and (3) the reinforcement type has no influence on flexural strength and modulus.

## 2. Materials and Methods

This study evaluated the flexural strength and flexural modulus of three different types of materials for provisional restorations: 3D-printed resin (NextDent C&B Micro Filled Hybrid, Vertex-Dental B.V., Soesterberg, The Netherlands), acrylic resin (Unifast LC, GC Corporation, Tokyo, Japan), and bis-acrylic resin (Luxatemp Fluorescence, DMG, GmbH, Hamburg, Germany). Additionally, for each material, four subgroups were created: no reinforcement, metal reinforcement (Stainless Steel Ortho-Flextech, Reliance Orthodontic Products, IL, USA), and glass fiber reinforcement, with either EverStick™ C&B or EverStick^TM^ NET (GC, Leuven, Belgium). A total of 150 samples were prepared, and all materials were mixed and polymerized according to the manufacturer’s instructions. Materials, chemical compositions, manufacturers, and lot numbers are displayed in Table 1.

**Figure 1:**
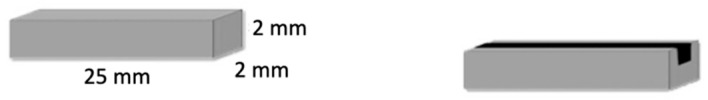
Specimen for control (**left**) and for reinforcement (**right**).

Printed specimens were designed into two shapes, a full rectangular bar (2 × 2 × 25 mm), according to ISO 4049:2019 [24], and a similar design with a central slot (1 × 1 mm) on one side, designed for reinforcement placement (Figure 1). Corresponding STL files were imported from the 3D Sprint Basic Software (v. 5.1, 3D Systems, Rock Hill, SC, USA). NextDent C&B Micro Filled Hybrid (NextDent 3D Systems, Vertex-Dental B.V., Netherlands) samples were printed using the NextDent 5100 3D Printer (NextDent 3D Systems, Vertex-Dental B.V., Netherlands) according to manufacturer recommendations regarding layer thickness and orientation. To ensure a correct impression and post-processing, the *z*-axis resolution was standardized.

Subsequent to the printing process, samples were used as molds to obtain conventional material specimen bars. An impression was performed using Aquasil Ultra LV (Dentsply Sirona, Konstanz, Germany) and Elite HD+ putty (Zhemarck, Rovigo, Italy), allowing for the acrylic and bis-acrylic to be injected directly into the silicone mold. A smooth, transparent plastic plate was used to press the new specimens over the silicone. For Unifast LC, samples were light-cured (Bluephase, Ivoclar Vivadent, Schaan, Liechtenstein, 1200 mW/cm^2^) for 10 s. After this initial polymerization, the specimens were then removed from the mold and polymerized for 20 s at four different points on both sides, in a total of 160 s. For Luxatemp, the self-curing process extended for 5 min, as recommended in the manufacturer’s instructions.

Regarding metal reinforcement, a stainless steel chain of 25 mm in length (Ortho-FlexTech 30″, Reliance Orthodontic, IL, USA) was placed directly into the slots. Furthermore, in the groups reinforced with EverStick C&B (GC, Leuven, Belgium), fiber bands were divided into two equal vertical segments in order to adjust the transverse volume to the existing slot, resorting to magnification and a microsurgical blade. Each segment was then carefully manipulated to form a cylindrical shape designed to fit precisely within the specimen slot. On the other hand, fiber reinforcement with EverStick NET (GC, Leuven, Belgium) required cutting fibers into two different fiber bands of the same size (25 mm), which were then wound to form a cylinder subsequently placed inside the slot.

Experimental groups are displayed in Table 2.

Finally, reinforcements were covered with the same material, with the exception of printed specimens where Unifast LC was employed. Afterwards, a smooth, transparent plastic plate was used to press the superficial layer, and for groups using Unifast LC, a similar polymerization protocol to the one described above was carried out. For Luxatemp, specimens were placed in a water bath at 60 °C for 5 min (Digital Water Bath, Nahita, Auxilab, S.L., Navarra, Spain) to ensure complete polymerization, according to the manufacturer’s recommendations. All specimens were wet-polished with a 1200-grit SiC paper (Hermes Schleifmittel GmbH, Hamburg, Germany).

Specimens were submitted to a three-point flexural strength test on a universal testing machine (Autograph, model AG-I, Shimadzu Corporation, Kyoto, Japan) with a span length of 20 mm, consisting of three rods with 2.0 mm in diameter, at a constant speed of 1 mm/min, according to ISO 4049:2019 [24]. Reinforcements were always facing down and resting on the lateral roll supports, where tensile stress is superior.

The flexure strength (*σ*), in MPa, and flexural modulus (*E*), in GPa, were calculated according to the following formulas at the maximum flexural load and on the load–displacement curve:

$$\sigma =\frac{3Fl}{2bh^{2}}\;E=\frac{Fl^{3}}{4dbh^{3}}$$

In the presented formulas, *F* represents the maximum load exerted on the specimen (Newtons), *l* is the distance between the supports (millimeters), *b* is the width at the center of the specimen measured immediately prior to testing (millimeters), *h* is the height at the center of the specimen measured immediately prior to testing (millimeters), and *d* represents the deflection at the load *F* (millimeters). Both values were obtained by using the TRAPEZIUM X v.38 software.

The statistical analysis was performed using RStudio (RStudio 2026.01.0+392 “Apple Blossom” Release). For each material property (flexural strength and flexural modulus), descriptive statistics, including means and standard deviations, were calculated. Normality Shapiro–Wilk and equal variance (Levene’s test) were used on the data sets prior to comparison. A two-way, non-parametric ANOVA using the aligned rank transform (ART) was employed for both flexural strength and flexural modulus results, considering both the fixed effects of reinforcement and resin, as well as the interaction between the two factors. Post hoc simple main effects were analyzed using pairwise Wilcoxon rank-sum tests on the raw data, with *p*-values adjusted using the Holm method to control multiple comparisons. The significance level for the statistical tests was 0.05.

## 3. Results

A total of 150 specimens were tested using the three-point flexural bending test. Data were visually analyzed and screened for extreme outliers, defined as values exceeding three times the interquartile range above the third quartile or below the first quartile within each experimental group. Two extreme outliers were identified (groups L/NET and N/metal) and excluded from subsequent analyses, as they likely represented physical defects in the individual test specimens rather than true material variance.

Verification of the assumptions of normality of distributions and homogeneity of variances found that there was an extreme violation of the assumption of the latter (*p* < 0.001 for the Levene test), and since sample size is not equal in all groups, a non-parametric approach, the ART tool (non-parametric ANOVA), was chosen. In this two-factor model, material and reinforcement were considered as fixed factors, including their interaction term (material × reinforcement).

Descriptive values of flexural strength are represented in Table 3.

For the flexural strength analysis, there was a statistically significant interaction between resin and reinforcement (F(6, 136) = 7.25, *p* < 0.001, partial η^2^ = 0.24), indicating that the flexural strength varies according to the combination of levels of material and reinforcement, indicating that the reinforcement type does not produce the same effect in all resins. In addition, there is also a main effect for material (F(2, 136) = 49.66, *p* < 0.001, partial η^2^ = 0.42) and for reinforcement (F(3, 136) = 83.67, *p* < 0.001, partial η^2^ = 0.65), with the latter accounting for 65% of the variance in the ranked flexural strength data.

**Figure 2:**
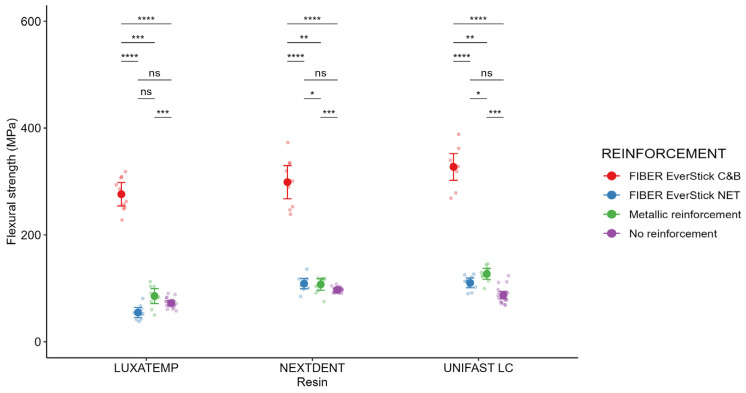
Mean flexural strength (MPa) of three provisional resin materials (Luxatemp, NextDent, UniFast LC) with different reinforcement strategies (EverStick C&B, EverStick NET, metallic reinforcement, and no reinforcement/control). Error bars represent 95% confidence intervals, with raw data points jittered in the background. Asterisks indicate statistically significant post hoc pairwise comparisons computed on aligned ranks. ns: *p* ≥ 0.05; * *p* < 0.05; ** *p* < 0.01; *** *p* < 0.001; **** *p* < 0.0001.

Wilcoxon post hoc pairwise comparisons on the flexural strength of each level of resin indicate that reinforcement with Fiber EverStick C&B results in statistically higher flexural strength than any other reinforcement method, regardless of the resin matrix to which it is applied, whereas reinforcement with Fiber EverStick NET is consistently non-different from the non-reinforced group, as detailed in Figure 2 and Table 4.

**Figure 3:**
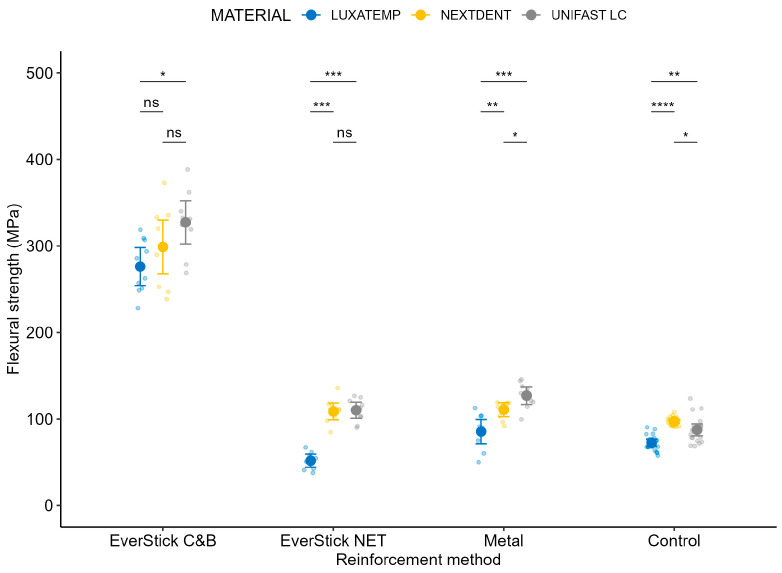
Mean flexural strength (MPa) of the three provisional resin materials (Luxatemp, NextDent, UniFast LC) within the four different reinforcement strategies (EverStick C&B, EverStick NET, metallic reinforcement and no reinforcement/control). Error bars represent 95% confidence intervals, with raw data points jittered in the background. Asterisks indicate statistically significant post hoc pairwise comparisons computed on aligned ranks. ns: *p* ≥ 0.05; * *p* < 0.05; ** *p* < 0.01; *** *p* < 0.001; **** *p* < 0.0001.

When considering the main effect reinforcement method, the resin contrasts indicate statistically significant differences between Luxatemp and all other resins, regardless of the reinforcement method used (Figure 3). This resin consistently presents lower flexural strength, whereas no statistically significant differences can be found between Nextdent and Unifast LC when reinforced with fibers. All contrasts are detailed in Table 5.

Similar results could be found for the flexural modulus variable (Table 6). The two-way ART ANOVA indicated a statistically significant interaction between resin and the reinforcement (F(6, 136) = 9.23, *p* < 0.001, partial η^2^ = 0.29), indicating that the flexural modulus is variable according to the combination of levels of resin and reinforcement. There is also a main effect for resin (F(2, 136) = 340.35, *p* < 0.001, partial η^2^ = 0.37) and for reinforcement (F(3, 136) = 147.79, *p* < 0.001, partial η^2^ = 0.77), both large effects. This indicates that there are statistically significant differences between resins regardless of the reinforcement method (main effect for resin) and that there are statistically significant differences between reinforcement methods regardless of the resin.

**Figure 4:**
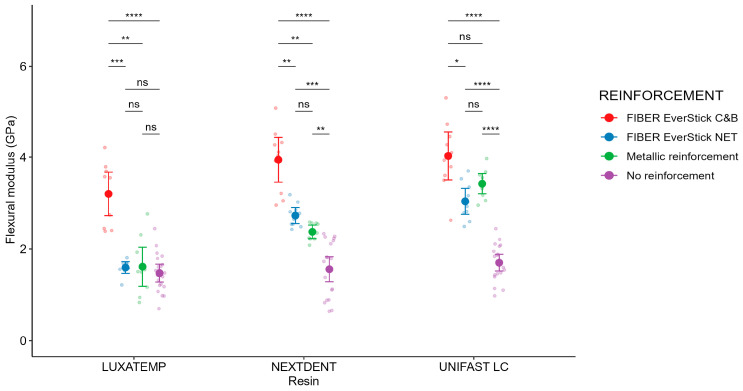
Mean flexural modulus (GPa) of the three provisional resin materials (Luxatemp, NextDent, UniFast LC) with different reinforcement strategies (EverStick C&B, EverStick NET, metallic reinforcement and no reinforcement/control). Error bars represent 95% confidence intervals, with raw data points jittered in the background. Asterisks indicate statistically significant post hoc pairwise comparisons computed on aligned ranks. ns: *p* ≥ 0.05; * *p* < 0.05; ** *p* < 0.01; *** *p* < 0.001; **** *p* < 0.0001.

When analyzing post hoc comparisons on the impact of reinforcement in each resin, it is interesting to notice that, in this case, reinforcement with any of the two fibers, Fiber EverStick C&B or Fiber EverStick NET, is not able to make the stiffness of Unifast LC higher than the reinforcement with metal (Figure 4). In addition, only the reinforcement with Fiber EverStick C&B is able to increase the flexural modulus of Luxatemp.

**Figure 5:**
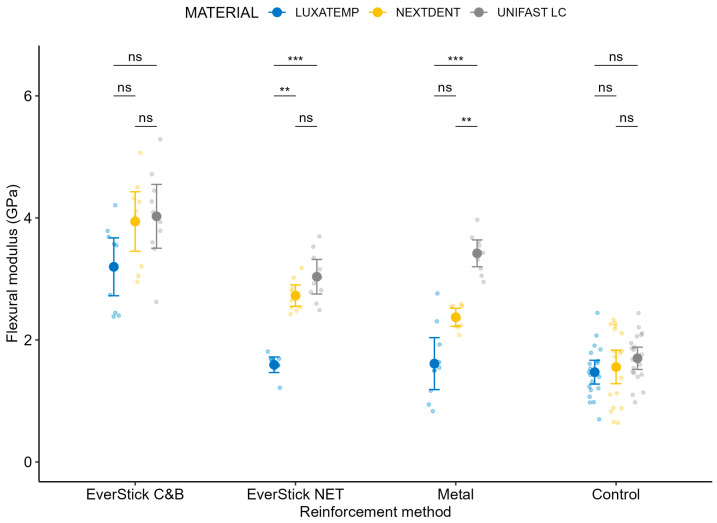
Comparison of mean flexural modulus (GPa) of the three provisional resin materials (Luxatemp, NextDent, UniFast LC) within the four different reinforcement strategies (EverStick C&B, EverStick NET, metallic reinforcement and no reinforcement/control). Error bars represent 95% confidence intervals, with raw data points jittered in the background. Asterisks indicate statistically significant post hoc pairwise comparisons computed on Wilcoxon-aligned ranks with Holm correction. ns: *p* ≥ 0.05; ** *p* < 0.01; *** *p* < 0.001.

Interestingly, there were no differences in the flexural modulus of the samples of non-modified resins and samples of resins reinforced with Fiber EverStick C&B (Figure 5).

Overall, the magnitude of the observed differences was relevant. In particular, fiber reinforcement with EverStick C&B produced extremely large effects on flexural strength across all tested materials. These findings indicate that the improvements associated with EverStick C&B were not only statistically significant but also substantial in magnitude and potentially clinically relevant.

Macroscopically, unreinforced specimens fractured into two separate parts, whereas all reinforced specimens remained connected after failure.

## 4. Discussion

This study was conducted according to ISO 4049:2019 [24], and it aimed to evaluate the flexural strength and modulus of different materials indicated for provisional restorations, either reinforced or not, with distinct materials. All null hypotheses, (1) there is no difference between the flexural strength and modulus of the tested materials, (2) the use of reinforcement has no influence on the flexural strength and modulus, and (3) the reinforcement type has no influence on flexural strength and modulus, were rejected, according to the obtained results.

Significant requirements such as superior mechanical properties, good biocompatibility, easy handling, and a good cost–benefit ratio must be considered when choosing materials for provisional restorations to predict a successful treatment [19,25,26]. Intraoral environment and occlusal loads, in the long term, are known to cause damage to provisional restorations [17,20]. Although in vitro studies do not completely simulate the conditions of the oral cavity, they allow a thorough understanding of materials and their expected behavior under a clinical situation [6]. Nevertheless, there are clinical factors, such as the presence of saliva and food/beverages, which constantly interact with materials exposed to the oral cavity [27].

Despite the increasing use of 3D-printed resins in clinical settings, there remains a lack of comparative data between these materials and conventional options like PMMA and bis-acrylics, particularly under reinforced and non-reinforced conditions [10,13,18]. This gap is clinically relevant considering the increased clinical use of additive manufacturing technologies and the demand for reliable provisional materials in extensive rehabilitations [10]. The values of flexural strength reported in the present study for the printed material are in agreement with the results of previous studies, justified by the presence of Micro Filled Hybrid (MFH) particles and the chemical adhesion between resin layers [2,28,29]. It should be noted that printing parameters and degree of conversion influence the microstructure and, consequently, the exhibited properties [20,30].

Regarding conventional provisional materials, in the unreinforced control groups, bis-acrylic resin (L) showed a statistically significantly lower flexural strength when compared to acrylic resin (U) (*p* = 0.012). Some studies reached the opposite conclusion [4,27], hypothesizing that bis-acrylic has an advantage because it contains multifunctional monomers, such as bisphenol A-glycidyl methacrylate (Bis-GMA) or triethylene glycol dimethacrylate (TEGDMA), which potentiate cross-linking interactions. Additionally, the associated mixing method of conventional materials leads to a higher chance of incorporating air bubbles and porosities, resulting in lower mechanical properties [30].

Due to the tendency for fracture of provisional restorations, particularly in areas with high occlusal stress or long-span partial dentures, the reinforcement of provisional restorations has been suggested. The literature indicates that the use of reinforcement contributes to the longevity of dental restorations, reducing the risk of chipping, wear, or fractures over time [20].

Fiber reinforcement stands out as one of the most extensively investigated strategies to address provisional restorations’ mechanical limitations [21]. This approach seems to significantly improve mechanical properties by interrupting crack propagation and dissipating internal stresses [6,21,22]. Among experimental groups, reinforcement with EverStick C&B rendered the highest flexural strength for the different resins. Unifast LC presented superior results (327.28 MPa), outperforming both NextDent (298.84 MPa) and Luxatemp (276.19 MPa). The combination of Luxatemp and EverStick NET yielded significantly lower flexural strength compared to the respective control group, which may be attributed to an enhanced tendency for void formation or incomplete resin impregnation in brittle materials such as bis-acrylic resins. Psarri et al. [7] argue that adequate adhesion between fibers and the reinforced material is the key to an increase in values of fracture resistance, and an effective impregnation procedure maximizes the contact between materials, which may explain the higher values of flexural strength obtained for Unifast LC, as in acrylic resin the presence of monomers enables the desired chemical adhesion. This is also consistent with findings of Hamza et al. [21] and Kamble et al. [31], who demonstrated that the incorporation of fibers in acrylic resins, such as PMMA, results in a significant increase in fracture resistance.

EverStick C&B is the only reinforcement method that successfully and significantly increased the stiffness of all three resins. EverStick C&B and EverStick NET differ primarily in their fiber structure, as the former consists of unidirectional glass fiber bundles, which provide high strength and rigidity along a single axis, while the latter is composed of a bidirectional woven fiber mesh, offering greater flexibility and adaptability but lower stiffness. Studies by Dyer et al. [22] and Vallittu et al. [32] reported that the incorporation of unidirectional fibers enhances structural integrity by controlling crack propagation and reducing deformation. The cross-sectional area occupied by the glass fiber should also be considered, as the size of the fiber may have a significant impact on the flexural strength and modulus. It is also noteworthy that the previously required management and adjustment of the fiber volume and its placement in the slot can influence this study’s results.

Furthermore, metallic reinforcement was able to exceed both the flexural strength and modulus values relative to the control group, although not always statistically significant. Whereas fibers achieve some chemical adhesion, metal reinforcement is purely based on mechanical interlocking, making the material more prone to debonding under load. Consequently, a more effective transference of stress to the fibers enables the increase in flexural and modulus values, while metal reinforcements tend to concentrate stress at the interface with the resin, increasing the risk of failure [7].

Fiber groups displayed greater dispersion of values, suggesting higher technique sensitivity, likely associated with fiber adaptation, impregnation, and interfacial bonding within the resin matrix. Additionally, the presence of scattered values and occasional outliers further supports the influence of specimen-level inconsistencies, such as void formation or incomplete wetting of the fiber network. In contrast, metallic reinforcement groups showed more clustered distributions, indicating more stable mechanical behavior and improved consistency of stress transfer. The non-reinforced groups generally exhibited lower variability but also lower median mechanical performance, reflecting a more homogeneous but mechanically weaker material response. Based on observations of the macroscopic behavior of samples during testing, non-reinforced samples fractured into two separate pieces. On the other hand, in the presence of reinforcement (fiber or metal), the samples did not separate into two pieces when the fracture occurred, indicating that mechanical connection was promoted by the reinforcement.

The findings of the present study reinforce that glass fiber reinforcement, particularly EverStick C&B, improves not only flexural strength but also stiffness, optimizing the mechanical behavior of provisional resins [6,21,22]. This improvement is particularly relevant in long-term provisional rehabilitations, where occlusal stability and resistance to deformation are essential [32,33]. Therefore, material selection should be based not only on average mechanical values but also on specific clinical conditions and the expected duration of use [7,33]. The improvements observed with EverStick C&B reinforcement were associated with extremely large effect sizes in all tested materials, indicating that these differences were not merely statistically significant but also mechanically meaningful. This is particularly relevant because *p*-values alone do not quantify the magnitude of improvement or its potential clinical relevance. The approximately threefold increase in flexural strength observed in several EverStick C&B groups suggests a substantial reinforcement effect, especially when compared with metal or EverStick NET reinforcement.

Although this study sought to provide scientifically grounded insights to support the clinical selection of more resistant and reliable provisional materials, further studies are necessary to understand the impact of some variables, such as the adverse conditions presented in the oral cavity, aging, and the use of different adhesive strategies, to complement current knowledge and guide standardization in future studies and clinical protocols.

This study presents some limitations that should be considered when interpreting the results. First, the absence of artificial aging protocols, such as thermal cycling, water storage, or fatigue loading, restricts the findings to the initial mechanical behavior of the materials and does not allow direct extrapolation to long-term clinical performance. Second, although efforts were made to standardize fiber placement and volume within the slot, some degree of operator-dependent variability cannot be completely excluded. The variability in the results may be attributed to several factors inherent to the experimental design as a result of technique sensitivity. Methodological aspects, such as interfacial bonding and the creation of hybrid material systems, should also be taken into account. Future research should incorporate failure behavior analysis and thermal and mechanical aging protocols to better simulate clinical conditions and assess the durability of these materials over time. Moreover, further studies evaluating different fiber configurations, placement techniques, and clinical scenarios would contribute to a more comprehensive understanding of their performance. Ultimately, well-designed clinical studies are necessary to confirm the in vitro findings and establish their relevance in daily practice.

## 5. Conclusions

Despite the inherent limitations of an in vitro study, the following conclusions can be drawn:

- Both material type and reinforcement strategy significantly influenced the flexural strength and modulus of provisional restorative materials.
- Fiber reinforcement with EverStick C&B provided the most consistent improvement in both flexural strength and stiffness across all tested materials. These improvements were associated with very large effect sizes, suggesting not only statistical significance but also substantial mechanical and potential clinical relevance.
- EverStick NET demonstrated limited or no beneficial effect and, in the bis-acryl resin, resulted in a significant reduction in flexural strength.
- Metal reinforcement led to moderate improvements in mechanical properties, with a less consistent effect when compared to fiber reinforcement.
- Reinforcement, regardless of type, altered the fracture pattern by preventing complete separation of specimens, suggesting improved structural integrity after failure.

## Figures and Tables

**Table 1:** Materials, chemical compositions, manufacturers, and lot numbers.

Material		Chemical Composition	Manufacturer	LOT
**3D Printed Provisional Resin**	NextDent C&B Micro Filled Hybrid	Methacrylate oligomer, methacrylate monomer, inorganic filler, phosphine oxides	NextDent, 3D Systems, Soesterberg, The Netherlands	WU101N01
**Acrylic Resin**	Unifast LC	Powder: ethyl–methyl methacrylate monomer, polymethylmethacrylate, barbituric acid derivative, organic copper compound, pigments Liquid: methyl methacrylate, N,N-dimethyl-ptoluidine trimethylolpropane, ethylene glycol dimethacrylate	GC Corporation, Tokyo, Japan	2307101
**Bis-acrylic resin**	Luxatemp Fluorescence	Dental glass, ethoxylated bisphenol A-dimethacrylate, aliph. Polyester diurethanes, unsaturated polyester resin, SiO_2_, additives Inorganic fillers: approx. 26 vol% (0.02–1.5 µm)	DMG GmbH, Hamburg, Germany	289474
**Fiber reinforced band**	EverStick™ C&B	Silane-treated, E-glass fibers (~61–65%), (1-methylethylidene)bis[4,1-phenyleneoxy(2-hydroxy-3,1-propanediyl)] bismethacrylate (25–50%), bis-GMA/PMMA semi-interpenetrating polymer network, 2-dimethylaminoethyl	GC, Leuven, Belgium	2505131
	EverStick^TM^ NET	(1-methylethylidene)bis[4,1-phenyleneoxy(2-hydroxy-3,1-propanediyl)], bismethacrylate, 2-dimethylaminoethyl methacrylate, ydroquinone		230705B
**Stainless Steel**	Stainless Steel Ortho-Flextech 30″	17–20% chromium, 8–12% nickel, 0.08–0.15% carbon and iron	Reliance Orthodontic, Itasca, IL, USA	122037

**Table 2:** Experimental groups.

Experimental Group	Material	Reinforcement	n
**U/control**	Unifast LC	Without reinforcement	20
**U/metal**		Metal	10
**U/C&B**		EverStick C&B	10
**U/NET**		EverStick NET	10
**N/control**	NextDent C&B	Without reinforcement	20
**N/metal**		Metal	10
**N/C&B**		EverStick C&B	10
**N/NET**		EverStick NET	10
**L/control**	Luxatemp Fluorescence	Without reinforcement	20
**L/metal**		Metal	10
**L/C&B**		EverStick C&B	10
**L/NET**		EverStick NET	10

**Table 3:** Descriptive statistics of flexural strength.

	Flexural Strength (MPa)								
Material	Reinforcement	n	Min	Max	Median	iqr	Mean	sd	se
LUXATEMP	FIBER EverStick C&B	10	228.22	318.63	274.22	51.23	276.19	30.68	9.70
LUXATEMP	FIBER EverStick NET	9	37.73	67.27	52.97	15.29	51.90	9.87	3.29
LUXATEMP	Metallic reinforcement	10	50.22	112.73	87.63	23.17	85.55	19.57	6.19
LUXATEMP	No reinforcement	20	57.83	90.47	72.63	9.14	72.63	8.90	1.99
NEXTDENT	FIBER EverStick C&B	10	238.59	373.01	299.41	67.85	298.84	43.48	13.75
NEXTDENT	FIBER EverStick NET	10	84.78	136.00	108.43	13.87	108.90	13.60	4.30
NEXTDENT	Metallic reinforcement	9	92.05	119.36	117.31	14.94	110.87	10.59	3.53
NEXTDENT	No reinforcement	20	91.17	107.99	98.28	6.87	97.43	4.86	1.09
UNIFAST LC	FIBER EverStick C&B	10	268.89	388.30	329.62	17.78	327.28	34.92	11.04
UNIFAST LC	FIBER EverStick NET	10	90.00	126.80	112.94	17.27	110.26	13.01	4.11
UNIFAST LC	Metallic reinforcement	10	99.61	145.84	126.86	16.98	127.13	14.30	4.52
UNIFAST LC	No reinforcement	20	68.91	123.81	85.75	15.00	87.54	14.93	3.34

**Table 4:** Pairwise Wilcoxon comparisons and effect sizes for the flexural strength of reinforcement strategies within resins.

Resin	Reinforcement A	Reinforcement B	Effect Size (r)	Effect Magnitude	*p*-Value (Holm)
LUXATEMP	FIBER EverStick C&B	FIBER EverStick NET	0.84	large	<0.001
	FIBER EverStick C&B	Metallic reinforcement	0.85	large	<0.001
	FIBER EverStick C&B	No reinforcement	0.80	large	<0.001
	FIBER EverStick NET	Metallic reinforcement	0.69	large	0.01
	FIBER EverStick NET	No reinforcement	0.73	large	<0.001
	Metallic reinforcement	No reinforcement	0.39	moderate	0.06
NEXTDENT	FIBER EverStick C&B	FIBER EverStick NET	0.85	large	<0.001
	FIBER EverStick C&B	Metallic reinforcement	0.84	large	<0.001
	FIBER EverStick C&B	No reinforcement	0.80	large	<0.001
	FIBER EverStick NET	Metallic reinforcement	0.19	small	0.45
	FIBER EverStick NET	No reinforcement	0.51	large	0.02
	Metallic reinforcement	No reinforcement	0.53	large	0.02
UNIFAST LC	FIBER EverStick C&B	FIBER EverStick NET	0.85	large	<0.001
	FIBER EverStick C&B	Metallic reinforcement	0.85	large	<0.001
	FIBER EverStick C&B	No reinforcement	0.80	large	<0.001
	FIBER EverStick NET	Metallic reinforcement	0.52	large	0.05
	FIBER EverStick NET	No reinforcement	0.61	large	0.01
	Metallic reinforcement	No reinforcement	0.76	large	<0.001

**Table 5:** Pairwise Wilcoxon comparisons and effect sizes for the flexural strength of resins within reinforcement strategies.

Reinforcement Strategy	Resin A	Resin B	Effect Size (r)	Effect Magnitude	*p*-Value (Holm)
FIBER EverStick C&B	LUXATEMP	NEXTDENT	0.25	small	0.57
	LUXATEMP	UNIFAST LC	0.68	large	0.01
	NEXTDENT	UNIFAST LC	0.30	moderate	0.57
FIBER EverStick NET	LUXATEMP	NEXTDENT	0.84	large	<0.001
	LUXATEMP	UNIFAST LC	0.84	large	<0.001
	NEXTDENT	UNIFAST LC	0.10	small	0.68
Metallic reinforcement	LUXATEMP	NEXTDENT	0.69	large	0.01
	LUXATEMP	UNIFAST LC	0.79	large	<0.001
	NEXTDENT	UNIFAST LC	0.62	large	0.02
No reinforcement	LUXATEMP	NEXTDENT	0.86	large	<0.001
	LUXATEMP	UNIFAST LC	0.55	large	<0.001
	NEXTDENT	UNIFAST LC	0.49	moderate	0.01

**Table 6:** Descriptive statistics of the flexural modulus.

	Flexural Modulus (GPa)								
Material	Reinforcement	n	Min	Max	Median	iqr	Mean	sd	se
LUXATEMP	FIBER EverStick C&B	10	2.38	4.21	3.38	1.14	3.20	0.66	0.21
LUXATEMP	FIBER EverStick NET	9	1.22	1.81	1.59	0.13	1.59	0.17	0.06
LUXATEMP	Metallic reinforcement	10	0.83	2.76	1.52	0.60	1.61	0.60	0.19
LUXATEMP	No reinforcement	20	0.70	2.44	1.45	0.49	1.47	0.42	0.09
NEXTDENT	FIBER EverStick C&B	10	2.95	5.07	4.07	0.92	3.94	0.68	0.22
NEXTDENT	FIBER EverStick NET	10	2.42	3.18	2.71	0.30	2.73	0.25	0.08
NEXTDENT	Metallic reinforcement	9	2.08	2.58	2.34	0.34	2.37	0.19	0.06
NEXTDENT	No reinforcement	20	0.64	2.33	1.64	1.08	1.56	0.58	0.13
UNIFAST LC	FIBER EverStick C&B	10	2.62	5.29	4.02	0.76	4.03	0.73	0.23
UNIFAST LC	FIBER EverStick NET	10	2.49	3.70	2.98	0.51	3.04	0.40	0.12
UNIFAST LC	Metallic reinforcement	10	2.95	3.97	3.45	0.39	3.42	0.31	0.10
UNIFAST LC	No reinforcement	20	0.98	2.44	1.72	0.52	1.70	0.39	0.09

## Data Availability

The original contributions presented in the study are included in the article, further inquiries can be directed to the corresponding author.

## References

[1] Shillingburg H.T., Sather D.A. (2012). Fundamentals of Fixed Prosthodontics.

[2] Jain S., Sayed M.E., Shetty M., Alqahtani S.M., Al Wadei M.H.D., Gupta S.G., Othman A.A.A., Alshehri A.H., Alqarni H., Mobarki A.H. (2022). Physical and Mechanical Properties of 3D-Printed Provisional Crowns and Fixed Dental Prosthesis Resins Compared to CAD/CAM Milled and Conventional Provisional Resins: A Systematic Review and Me-ta-Analysis. Polymers.

[3] Mârțu I., Murariu A., Baciu E.R., Savin C.N., Foia I., Tatarciuc M., Diaconu-Popa D. (2022). An Interdisciplinary Study Regarding the Characteristics of Dental Resins Used for Tem-porary Bridges. Medicina.

[4] Haselton D.R., Diaz-Arnold A.M., Vargas M.A. (2002). Flexural strength of provisional crown and fixed partial denture resins. J. Prosthet. Dent..

[5] Alzahrani S.J., Hajjaj M.S., Azhari A.A., Ahmed W.M., Yeslam H.E., Carvalho R.M. (2023). Mechanical Properties of Three-Dimensional Printed Provisional Resin Materials for Crown and Fixed Dental Prosthesis: A Systematic Review. Bioengineering.

[6] Orsi I.A., Soares R.G., Villabona C.A., Panzeri H. (2012). Evaluation of the flexural strength and elastic modulus of resins used for temporary restorations reinforced with particulate glass fibre. Gerodontology.

[7] Psarri C., Kourtis S. (2020). Effect of fiber-reinforcement on the strength of polymer materials for provisional restorations: An in vitro study. J. Esthet. Restor. Dent..

[8] Padunglappisit C., Suwanprateep N., Chaiwerawattana H., Naruphontjirakul P., Panpisut P. (2023). An in vitro assessment of biaxial flexural strength, degree of monomer conversion, color stability, and ion release in provisional restorations containing Sr-bioactive glass nanoparticles. Biomater. Investig. Dent..

[9] Young H.M., Smith C.T., Morton D. (2001). Comparative in vitro evaluation of two provisional restorative materials. J. Prosthet. Dent..

[10] Bae E.J., Jeong I.D., Kim W.C., Kim J.H. (2017). A comparative study of additive and subtractive manufacturing for dental restorations. J. Prosthet. Dent..

[11] Tahayeri A., Morgan M., Fugolin A.P., Bompolaki D., Athirasala A., Pfeifer C.S., Ferracane J.L., Bertassoni L.E. (2018). 3D printed versus conventionally cured provisional crown and bridge dental materials. Dent. Mater..

[12] Kihara H., Sugawara S., Yokota J., Takafuji K., Fukazawa S., Tamada A., Hatakeyama W., Kondo H. (2021). Applications of three-dimensional printers in prosthetic dentistry. J. Oral Sci..

[13] Digholkar S., Madhav V.N.V., Palaskar J. (2016). Evaluation of the flexural strength and microhardness of provisional crown and bridge materials fabricated by different methods. J. Indian Prosthodont. Soc..

[14] Juntavee N., Juntavee A., Srisontisuk S. (2023). Flexural Strength of Various Provisional Restorative Materials for Rehabilitation After Aging. J. Prosthodont..

[15] Saini R.S., Gurumurthy V., Quadri S.A., Bavabeedu S.S., Abdelaziz K.M., Okshah A., Alshadidi A.A.F., Yessayan L., Mosaddad S.A., Heboyan A. (2024). The flexural strength of 3D-printed provisional restorations fabricated with different resins: A systematic review and meta-analysis. BMC Oral Health.

[16] Souza A.L.C., Filho J.C., Rocha S.S.D. (2023). Flexural strength and Vickers hardness of milled and 3D-printed resins for pro-visional dental restorations. Braz. J. Oral Sci..

[17] Ellakany P., Fouda S.M., Mahrous A.A., AlGhamdi M.A., Aly N.M. (2022). Influence of CAD/CAM Milling and 3D-Printing Fabrication Methods on the Mechanical Properties of 3-Unit Interim Fixed Dental Prosthesis after Thermo-Mechanical Aging Process. Polymers.

[18] Sivakorn S., Lin R., Park C. (2026). Effect of Fiber Reinforcement on the Flexural Strength of Long-Span, 3D-Printed, Interim Fixed Dental Prostheses. Int. J. Prosthodont..

[19] Raszewski Z., Kulbacka J., Nowakowska-Toporowska A. (2022). Mechanical Properties, Cytotoxicity, and Fluoride Ion Release Capacity of Bioactive Glass-Modified Methacrylate Resin Used in Three-Dimensional Printing Technology. Materials.

[20] Alshamrani A., Alhotan A., Kelly E., Ellakwa A. (2023). Mechanical and Biocompatibility Properties of 3D-Printed Dental Resin Reinforced with Glass Silica and Zirconia Nanoparticles: In Vitro Study. Polymers.

[21] Hamza T.A., Rosenstiel S.F., El-Hosary M.M., Ibraheem R.M. (2006). Fracture Resistance of Fiber-Reinforced PMMA Interim Fixed Partial Dentures. J. Prosthodont..

[22] Dyer S. (2004). Effect of fiber position and orientation on fracture load of fiber-reinforced composite. Dent. Mater..

[23] Garoushi S., Vallittu P.K., Lassila L.V.J. (2007). Short glass fiber reinforced restorative composite resin with semi-inter penetrating polymer network matrix. Dent. Mater..

[24] (2019). Dentistry—Polymer-Based Restorative Materials.

[25] Rodríguez-Guardado W.E., Rivera-Muñoz E.M., Serrano-Bello J., Alvarez-Perez M.A., Domínguez-Pérez R.A., Salmerón-Valdés E.N., Vázquez F.C.V., Chanes-Cuevas O.A., Millán-Malo B., Peza-Ledesma C.L. (2024). Physical and structural characterization of bis-acryl com-posite resin. Sci. Rep..

[26] Singh A., Garg S. (2016). Comparative Evaluation of Flexural Strength of Provisional Crown and Bridge Materials-An Invitro Study. J. Clin. Diagn. Res..

[27] Nejatidanesh F., Momeni G., Savabi O. (2009). Flexural Strength of Interim Resin Materials for Fixed Prosthodontics. J. Prosthodont..

[28] Park S.M., Park J.M., Kim S.K., Heo S.J., Koak J.Y. (2020). Flexural Strength of 3D-Printing Resin Materials for Provisional Fixed Dental Prostheses. Materials.

[29] Choi Y., Moon W., Manso A.P., Park Y.S., Lim B.S., Chung S.H. (2024). Shear bond strength of orthodontic brackets bonded with pri-mer-incorporated orthodontic adhesives and unpolymerized 3-dimensional printing materials on 3-dimensional-printed crowns. Am. J. Orthod. Dentofac. Orthop..

[30] Dixon D.L., Fincher M., Breeding L.C., Mueninghoff L.A. (1995). Mechanical properties of a light-polymerizing provisional restorative material with and without reinforcement fibers. J. Prosthet. Dent..

[31] Kamble V.D., Parkhedkar R.D., Mowade T.K. (2012). The effect of different fiber reinforcements on flexural strength of provisional re-storative resins: An in-vitro study. J. Adv. Prosthodont..

[32] Vallittu P.K. (1999). Flexural properties of acrylic resin polymers reinforced with unidirectional and woven glass fibers. J. Prosthet. Dent..

[33] Abu-Obaid A.I., Alotaibi A.M., Binmeqren A.F., Albarrak R.A., Albaqami M.S., Alshahrani A.S. (2024). Comparison of the Flexural Strength and Elastic Modulus of Conventional, Milled and 3D-Printed Interim Restorative Materials Subjected to Different Intervals of Accelerated Aging. Open Access Libr..

